# Natural History of Nonmetastatic Prostate Cancer Managed With Watchful Waiting

**DOI:** 10.1001/jamanetworkopen.2024.14599

**Published:** 2024-06-04

**Authors:** Eugenio Ventimiglia, Rolf Gedeborg, Johan Styrke, David Robinson, Pär Stattin, Hans Garmo

**Affiliations:** 1 Division of Experimental Oncology/Unit of Urology, IRCCS Ospedale San Raffaele, Milan, Italy; 2 Department of Surgical Sciences, Uppsala University, Uppsala, Sweden; 3 Medical Products Agency, Uppsala, Sweden; 4 Department of Surgical and Perioperative Sciences, Urology and Andrology, Umeå University, Umeå, Sweden; 5 Department of Urology, Umeå University, Region of Jönköping, Sweden

## Abstract

**Importance:**

It is uncertain to what extent watchful waiting (WW) in men with nonmetastatic prostate cancer (PCa) and a life expectancy of less than 10 years is associated with adverse consequences.

**Objective:**

To report transitions to androgen deprivation therapy (ADT), castration-resistant prostate cancer (CRPC), death from PCa, or death from other causes in men treated with a WW strategy.

**Design, Setting, and Participants:**

This nationwide, population-based cohort study included men with nonmetastatic PCa diagnosed since 2007 and registered in the National Prostate Cancer Register of Sweden with WW as the primary treatment strategy and with life expectancy less than 10 years. Life expectancy was calculated based on age, the Charlson Comorbidity Index (CCI), and a drug comorbidity index. Observed state transition models complemented observed data to extend follow-up to more than 20 years. Analyses were performed between 2022 and 2023.

**Exposure:**

Nonmetastatic PCa.

**Main Outcomes and Measures:**

Transitions to ADT, CRPC, death from PCa, and death from other causes were measured using state transition modeling.

**Results:**

The sample included 5234 men (median [IQR] age at diagnosis, 81 [79-84] years). After 5 years, 954 men with low-risk PCa (66.2%) and 740 with high-risk PCa (36.1%) were still alive and not receiving ADT. At 10 years, the corresponding proportions were 25.5% (n = 367) and 10.4% (n = 213), respectively. After 10 years, 59 men with low-risk PCa (4.1%) and 221 with high-risk PCa (10.8%) had transitioned to CRPC. Ten years after diagnosis, 1330 deaths in the low-risk group (92.3%) and 1724 in the high-risk group (84.1%) were from causes other than PCa.

**Conclusions and Relevance:**

These findings suggest that the WW management strategy is appropriate for minimizing adverse consequences of PCa in men with a baseline life expectancy of less than 10 years.

## Key Points

**Question:**

Is watchful waiting (WW) an appropriate management strategy in men with nonmetastatic prostate cancer and life expectancy of less than 10 years?

**Findings:**

In this cohort study of 5234 men diagnosed with nonmetastatic prostate cancer and a life expectancy of less than 10 years at diagnosis, WW was associated with a low risk of cancer progression and mortality.

**Meaning:**

These findings suggest that WW is an appropriate treatment strategy for men with nonmetastatic prostate cancer and life expectancy of less than 10 years.

## Teaser

This cohort study examines whether a watchful waiting treatment strategy is associated with adverse outcomes in men with nonmetastatic prostate cancer and a life expectancy of less than 10 years in Sweden.

## Introduction

Men with low- or intermediate-risk prostate cancer (PCa) have a low risk of progression and virtually no risk of death from PCa during the first 10 years after diagnosis.^1^ For men with a life expectancy shorter than 10 years, current guidelines recommend that these individuals are treated with watchful waiting (WW).^2^

The purpose of WW in PCa is to avoid adverse effects of active treatment in men who are unlikely to experience long-term benefits of such treatment due to their limited life expectancy. The desirable overall outcome of WW in these men is to maximize the person-time spent without androgen deprivation therapy (ADT) and minimize time with castration-resistant PCa (CRPC) and associated debilitating symptoms of PCa while at the same time maximizing overall survival.^2^

It is therefore important to describe the outcomes of WW at the population level in order to inform clinicians about appropriate patient selection and treatment strategies. Most data in support of WW for low- and intermediate-risk PCa come from small historical studies performed at tertiary referral centers.^3,4^ These series include men diagnosed more than 40 years ago and may not be representative for men with a current diagnosis. These historical studies usually focused on death as the event and did not describe the disease trajectory leading up to death.

Our group has previously developed 2 state transition models. One describes PCa disease trajectories, including changes in treatment strategy and outcomes up to 30 years after diagnosis,^5^ and the other estimates life expectancy in the general population.^6^ The aims of this study were to describe the distribution of remaining lifetime spent without and with ADT, progression to CRPC, time to death, and cause of death among men with PCa primarily treated with WW and to confirm that WW remains a safe and beneficial treatment strategy for these men.

## Methods

### Data Sources

This cohort study used data from the National Prostate Cancer Register (NPCR) of Sweden, which captures 98% of all incident PCa cases compared with the Swedish National Cancer Register to which reporting is mandated by law.^7^ In the Prostate Cancer Database Sweden, NPCR data have been enriched with data from other registers, including the Patient Register, the Cause of Death Register, and the Prescribed Drug Register, by use of the Swedish person identity number as previously described in detail.^8^ For each man with PCa, 5 control men free of PCa were selected from the Swedish general population, matching age and county of residence. This study was approved by the regional ethical review board of Uppsala University. As in all Swedish clinical cancer registers and other quality registers, all men with PCa are informed at time of diagnosis and treatment that they will be included in Sweden’s NPCR unless they opt out; therefore, written informed consent was not required. This study followed the Strengthening the Reporting of Observational Studies in Epidemiology (STROBE) reporting guideline.

The Patient Register includes information on all in-hospital care since 1987 and all outpatient specialist care since 2001. During the study period, diagnoses were recorded according to the Swedish modification of the *International Statistical Classification of Diseases, Tenth Revision* (*ICD-10*). The Charlson Comorbidity Index (CCI) was calculated based on *ICD-10* codes registered as a primary or secondary diagnosis in the Patient Register beginning 10 years before the start of follow-up.^9^ We excluded *ICD-10* codes for PCa (C61), and metastases (C77-C80) if they were registered in conjunction with C61, in line with previous adaptations of CCI for cancer studies.^9^ Information on orchidectomy was also retrieved from the Patient Register.

The Cause of Death Register contains the date and cause of death of all Swedish residents since 1952. The Prescribed Drug Register contains detailed information on all prescriptions dispensed in Sweden since July 1, 2005. This register includes filled prescriptions but not medicines sold over the counter or drugs administered during inpatient care. Filled prescriptions were used to define initiation of ADT and to calculate a drug comorbidity index (DCI).^10,11^

### Study Population

We identified men diagnosed between January 1, 2007, and December 31, 2019, with nonmetastatic PCa and with WW registered as primary treatment strategy in NPCR (eFigure 1 in Supplement 1).^12,13,14^ Men with stage cT4, cN1, M1, and/or prostate-specific antigen level greater than 100 ng/mL were excluded. The European Association of Urology guidelines recommend WW for men with PCa who have a life expectancy less than 10 years. We therefore only included men with an estimated life expectancy less than 10 years (described later). Data on race and ethnicity are not routinely collected in the Prostate Cancer Database Sweden and are not reported. The WW-specific risk categories were defined based on a modified version of the National Comprehensive Cancer Network risk categorization (eTable in Supplement 1). Men who received curative treatment within 2 years of diagnosis were considered to have had an unclear treatment strategy and were excluded from analysis since the state transition model used did not include this category.^5,8^

### Follow-Up

Follow-up started at the date of PCa diagnosis and ended at the date of death or last date of follow-up. The observed follow-up time was censored at the last available date of prescription of ADT in the Prescribed Drug Register, which was at the latest December 31, 2020, for 95% of the study population. Men registered with WW and who received curative treatment more than 2 years after diagnosis were censored on the date of curative treatment.

### Estimation of Life Expectancy

Life expectancy was calculated based on age, CCI, and DCI. To calculate the CCI, we used information in the Patient Register and Swedish National Cancer Register on discharge diagnoses during a 10-year look-back period.^15^ The DCI was calculated based on filled prescriptions registered in the Prescribed Drug Register during 365 days prior to the date of diagnosis.^10,11^

The control men free of PCa were used to estimate life expectancy. Both the CCI and DCI were calculated at the start of follow-up and updated each consecutive year until the end of follow-up. Microsimulations based on combinations of CCI (incrementally from 0 to 13) and discrete levels of age (incrementally from 50 years to 99 years) and DCI (incrementally by 0.25 from −0.75 to 13.50) were performed to estimate life expectancy, as previously described.^6^

These simulation results were applied to each man in the study population based on his observed age, CCI, and DCI at the date of diagnosis. The actual life expectancy was retrieved from an interpolation between the closest discrete levels of age and DCI in the above microsimulations.

### State Transitions During Follow-Up

**Figure 1.**
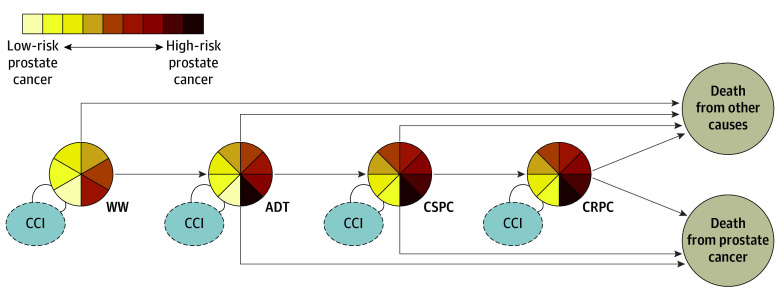
States and Transitions Defined in the State Transition Model Arrows indicate transitions, and circles indicate states. Pie charts with multicolored slices represent transient stages (watchful waiting [WW], androgen deprivation therapy [ADT], castration-sensitive prostate cancer [CSPC], and castration-resistant prostate cancer [CRPC]), and beige circles represent absorbing states. The colors in transient states indicate disease severity categories at date of entry to the state. The gray circles represent additional information considered to facilitate the estimation of transition probabilities (Charlson Comorbidity Index [CCI]).

We considered transitions to ADT, CRPC, death from PCa, or death from other causes (Figure 1). A transition to CRPC required that the man be considered castrated. This point was defined as the earliest date reflecting administration of 90 defined daily doses of a gonadotropin-releasing hormone agonist (Anatomical Therapeutical Chemical code L02AE) or at the date of orchiectomy.

The transition date to CRPC is not registered in any register in Sweden. Therefore, we captured transitions to this state indirectly based on our previously described state transition model.^16^ This model uses discrete 28-day time steps to describe state transitions.

For each man, the remaining follow-up time was split into 28-day time steps. At each time step, observed changes in CCI were recorded. If the end of follow-up was death, the cause of death was identified.

The probability for transition to CRPC at each time step was then calculated based on the state transition model (eFigure 2 in Supplement 1).^17^ The probability for no transition to CRPC was also estimated by this model. Among the potential dates for transition to CRPC, 1 was randomly selected proportional to the probability distribution defined by the model. The eMethods in Supplement 1 provide additional details.

### Microsimulation After the Censoring Date

To increase follow-up time up to 20 years, state transitions before censoring were based on observed data and then combined with simulated data in the nonobserved period. The simulation was based on age, state-specific PCa risk category, history of changes in CCI, current CCI, and treatment history at the date of censoring.

The microsimulation was performed as previously described,^5,9,17,18^ and further details are provided in the eMethods and eFigure 2 in Supplement 1. To increase precision, 100 replicates of each man were simulated.

### Statistical Analysis

The data analysis was performed between 2022 and 2023. From the combined observed and simulated patient states over time, we visualized proportions of disease states after 5, 10, 15, and 20 years of follow-up using Sankey diagrams stratified for PCa risk group at diagnosis. We estimated the proportions of men who died of PCa, received ADT, or reached the CRPC state and the proportion of lifetime receiving ADT compared with life expectancy, using separate regression models for each PCa risk category. Estimated and observed outcomes over the range of life expectancy are described. Life expectancy was modeled as a natural cubic spline with inner knots at the 35th and 65th percentile of life expectancy and boundary knots in the 2.5th and 97.5th percentiles of life expectancy.

We assumed that lower risk of PC death and long survival without curative treatment represented the largest benefit of WW. To illustrate this, we plotted the proportion of men who died of PCa vs the proportion of lifetime spent without curative PCa treatment. Statistical analyses were performed using RStudio, version 0.98 for R, version 3.0.2 (R Foundation for Statistical Computing).

## Results

### Patient Characteristics

We studied 5234 men diagnosed between January 1, 2007, and December 31, 2019, with nonmetastatic PCa with a registered treatment strategy of WW. Median age at diagnosis was 81 years (IQR, 79-84 years), and life expectancy was less than 6 years in 1195 men (22.8%) (Table). A total of 2050 men (39.2%) had a high-risk cancer.

**Table.** Baseline Characteristics of Men Diagnosed With Nonmetastatic Prostate Cancer in 2007 to 2019 and Registered in National Prostate Cancer Register of Sweden as Primarily Watchful Waiting^a^

Characteristic	No. of patients (%)			
	Low risk (n = 1441)	Intermediate risk (n = 1743)	High risk (n = 2050)	All (N = 5234)
Age, median (IQR), y	80.8 (78.2-83.2)	81.1 (78.6-83.3)	82.2 (79.6-85.0)	81.4 (78.8-84.0)
T stage				
T1a	700 (48.6)	51 (2.9)	10 (0.5)	761 (14.5)
T1b	NA	343 (19.7)	140 (6.8)	483 (9.2)
T1c	741 (51.4)	786 (45.1)	162 (7.9)	1689 (32.3)
T2	NA	563 (32.3)	1277 (62.3)	1840 (35.2)
T3	NA	NA	461 (22.5)	461 (8.8)
M stage				
M0	840 (58.3)	1059 (60.8)	1245 (60.7)	3144 (60.1)
MX	601 (41.7)	684 (39.2)	805 (39.3)	2090 (39.9)
Gleason score				
2-6	1302 (90.4)	924 (53.0)	225 (11.0)	2451 (46.8)
7 (3 + 4)	110 (7.6)	585 (33.6)	795 (38.8)	1490 (28.5)
7 (4 + 3)	NA	161 (9.2)	462 (22.5)	623 (11.9)
8	NA	17 (1.0)	299 (14.6)	316 (6.0)
9-10	NA	NA	102 (5.0)	102 (1.9)
Missing	29 (2.0)	56 (3.2)	167 (8.1)	252 (4.8)
Prostate-specific antigen, ng/mL^b^				
0-10.0	764 (53.0)	831 (47.7)	705 (34.4)	2300 (43.9)
10.1-20.0	404 (28.0)	523 (30.0)	618 (30.1)	1545 (29.5)
20.1-50.0	19 (1.3)	243 (13.9)	546 (26.6)	808 (15.4)
>50.0	NA	23 (1.3)	114 (5.6)	137 (2.6)
Missing	254 (17.6)	123 (7.1)	67 (3.3)	444 (8.5)
Mode of detection				
Screening	315 (21.9)	546 (31.3)	736 (35.9)	1597 (30.5)
Lower urinary tract symptoms	843 (58.5)	843 (48.4)	800 (39.0)	2486 (47.5)
Other symptoms	254 (17.6)	316 (18.1)	463 (22.6)	1033 (19.7)
Missing	29 (2.0)	38 (2.2)	51 (2.5)	118 (2.3)
Charlson Comorbidity Index				
0	553 (38.4)	737 (42.3)	936 (45.7)	2226 (42.5)
1	297 (20.6)	370 (21.2)	428 (20.9)	1095 (20.9)
2	326 (22.6)	344 (19.7)	370 (18.0)	1040 (19.9)
>2	265 (18.4)	292 (16.8)	316 (15.4)	873 (16.7)
Life expectancy, y^c^				
<6	307 (21.3)	335 (19.2)	553 (27.0)	1195 (22.8)
6-8	463 (32.1)	562 (32.2)	741 (36.1)	1766 (33.7)
>8	671 (46.6)	846 (48.5)	756 (36.9)	2273 (43.4)

Abbreviation: NA, not applicable.

^a^ Modified National Comprehensive Cancer Network criteria as specified in the eTable in Supplement 1.

^b^ To convert prostate-specific antigen to µg/L, multiply by 1.

^c^ Life expectancy based on age, Charlson Comorbidity Index, and the drug comorbidity index. Restricted to men with a life expectancy of less than 10 years and stratified by risk group.

### Treatment With ADT

**Figure 2.**
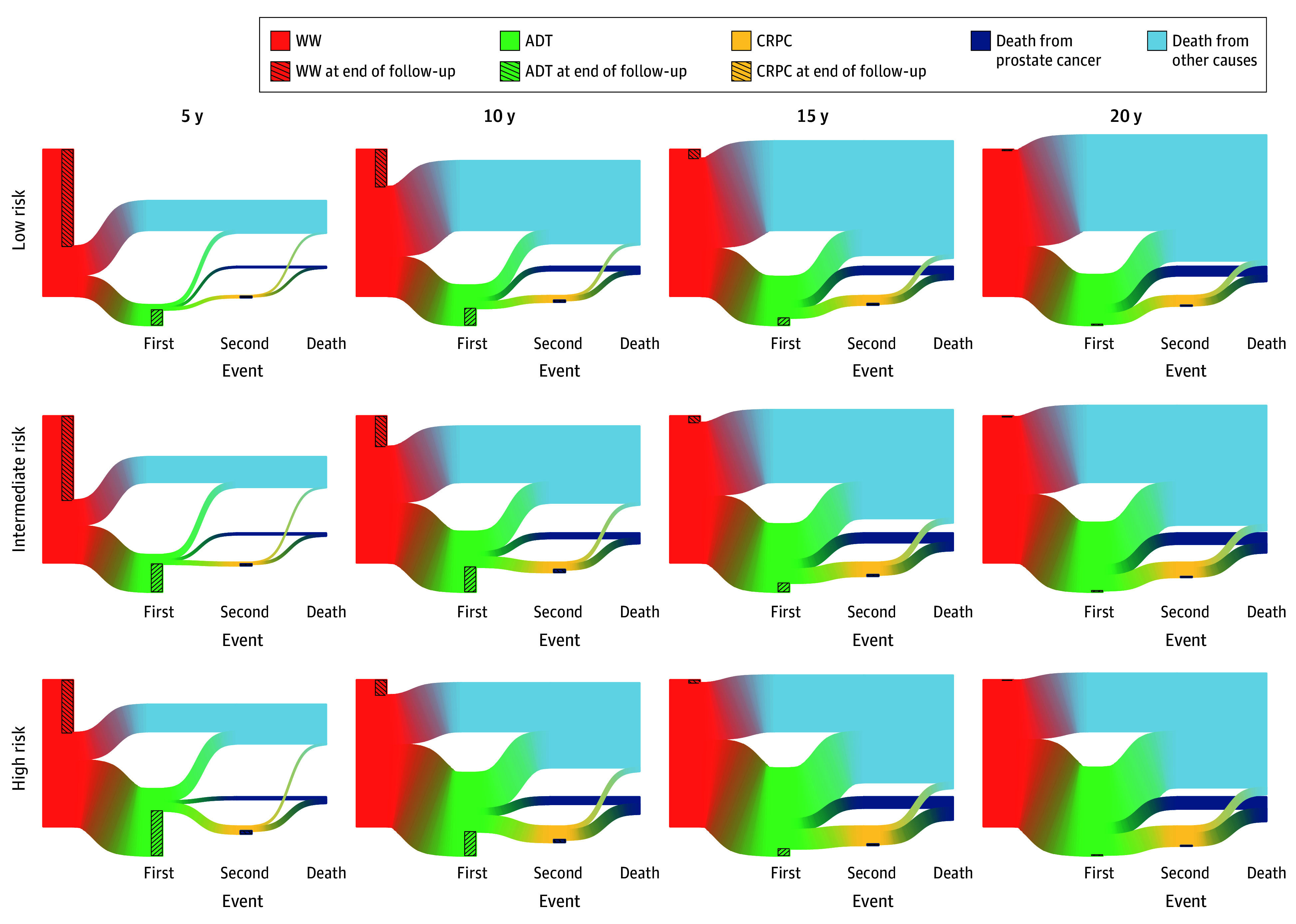
States and State Transitions During Follow-Up for Men With Prostate Cancer Treated With a Watchful Waiting (WW) Strategy The first event represents the transtion from a WW strategy to treatment with androgen deprivation therapy (ADT); the second event represents the development of castration-resistant prostate cancer (CRPC).

**Figure 3.**
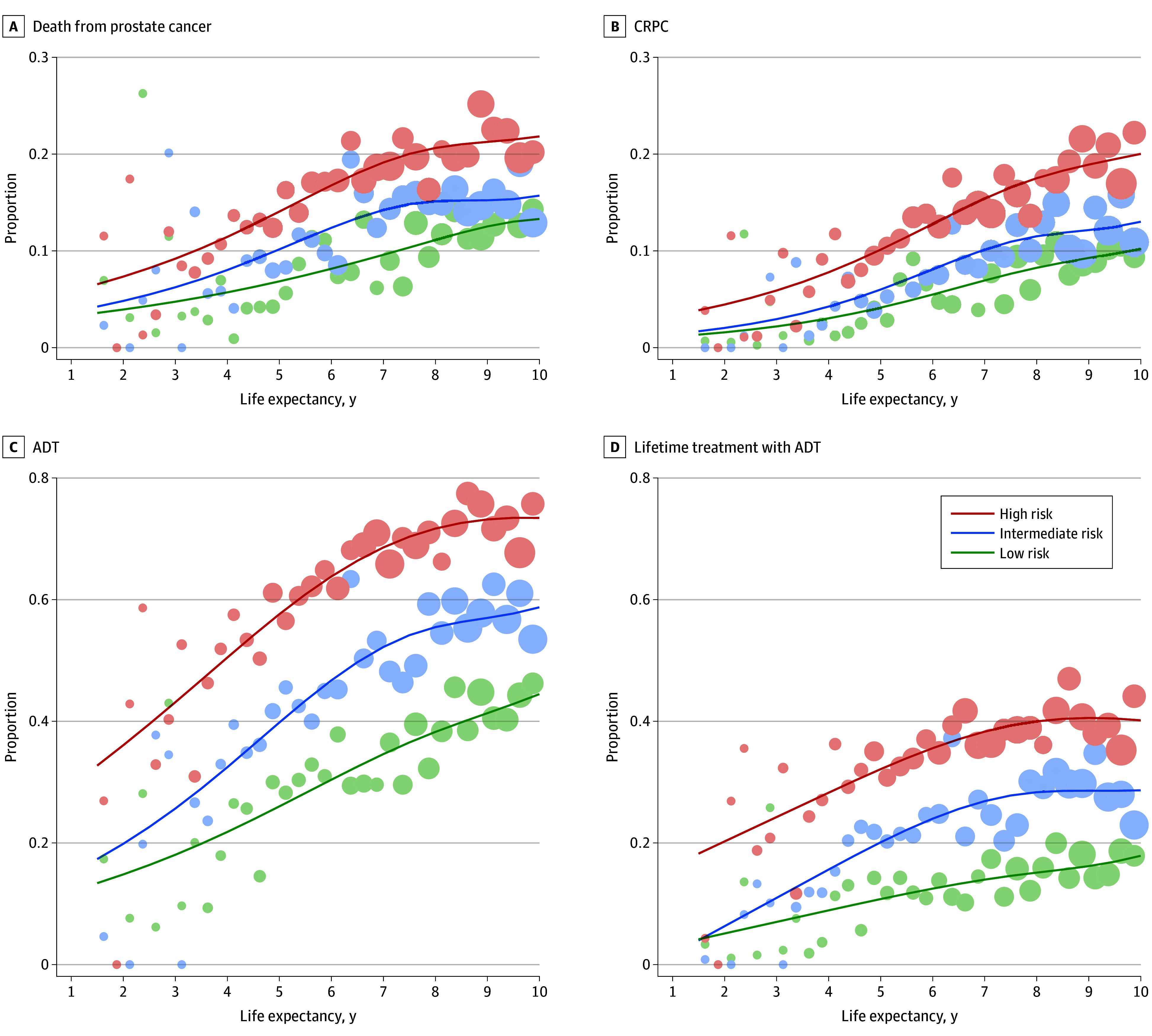
Graphical Association Between Oncologic Outcomes of Men Treated With Watchful Waiting and Expected Remaining Lifetime at Diagnosis The size of the circles is proportional to the number of men (smallest circles represent 1-10 men; largest circles represent 121-130 men). ADT indicates androgen deprivation therapy; CRPC, castration-resistant prostate cancer.

After 5 years, 954 men (66.2%) with low-risk PCa and 740 (36.1%) with high-risk PCa were still alive and did not require ADT (Figure 2). At 10 years, the corresponding proportions were 25.5% (n = 367) and 10.4% (n = 213), respectively. The proportion of men who did not require ADT and the proportion of remaining lifetime without ADT decreased nonlinearly with increasing life expectancy (Figure 3). A total of 32 men (44.4%) with low-risk PCa and 10-year life expectancy transitioned to ADT compared with 60 (73.5%) with high-risk disease.

### Progression to CRPC

After 10 years, 59 men (4.1%) in the low-risk group and 221 (10.8%) in the high-risk group had transitioned to CRPC (Figure 2). After 20 years, the corresponding proportions were 6.7% (97 men) and 13.3% (273 men). The proportion of men who progressed to CRPC increased with increasing life expectancy (Figure 3). Of men with a 10-year life expectancy, 10.0% in the low-risk category and 20.0% in the high-risk category progressed to CRPC.

### Death

After 10 years, 1330 men (92.3%) in the low-risk group and 1724 (84.1%) in the high-risk group died of causes other than PCa (Figure 2). Similarly to CRPC, the proportion of deaths from PCa increased with increasing life expectancy (Figure 3).

### Treatment-Free Survival

**Figure 4.**
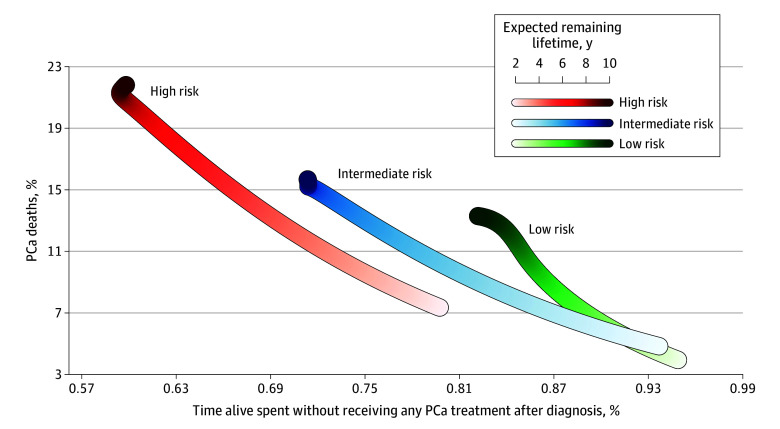
Association Between Proportion of Life-Years Without Receiving Any Prostate Cancer (PCa) Treatment After Diagnosis and Risk of Death From PCa

Men with low- or intermediate-risk PCa were able to spend most of their remaining lifetime without receiving ADT and had a low risk of death from PCa (Figure 4; eFigure 3 in Supplement 1). The proportion of remaining lifetime without receiving ADT was 82.1% for men with low-risk PCa and a 10-year life expectancy. Their risk of death from PCa was 13.3%. Although 21.8% with high-risk PCa and 10-year life expectancy died of PCa, they were still able to spend 59.8% of their remaining lifetime without receiving ADT.

## Discussion

In this nationwide, population-based cohort study, we combined observed data with state transition models to describe a complete follow-up of 5234 men with nonmetastatic PCa and less than 10 years’ life expectancy who were treated with a WW strategy. The results confirm that WW is an appropriate treatment strategy in older men with nonmetastatic PCa given that the goal is to maximize person-time spent without ADT and minimize time with CRPC while at the same time maximizing overall survival.

Overall, most men spent the larger part of their remaining lifetime without ADT, and most deaths were attributable to causes other than PCa. Androgen deprivation therapy is associated with decreased quality of life, making the lifetime spent without ADT an important aspect of WW.^19^ In addition, ADT has clinically relevant adverse effects, which not only impair quality of life but also increase comorbidity and possibly reduce life expectancy.^20^

As expected, more advanced cancer at diagnosis was associated with an earlier start of ADT, shorter lifetime spent without ADT, progression to CRPC, and higher risk of death from PCa. However, even in high-risk men, one-third had not initiated ADT after 5 years, and after 10 years, only a small proportion had transitioned to CRPC. In all risk groups, only a small proportion of deaths were attributed to PCa. The higher proportion of deaths from PCa in men with high-risk cancer suggests that other primary treatment strategies could be considered; however, this should be weighed against possible adverse effects. These results may provide reassurance to men who choose WW as a treatment strategy for PCa since a large proportion of them may not experience cancer progression and might die of other causes.

This large, nationwide, population-based study provides complete follow-up of men with PCa treated with a WW strategy. The only way to achieve long-term follow-up in the current era is to combine observed data with appropriate modeling strategies. We therefore used previously validated simulation models for PCa disease trajectories^17,18^ and estimations of life expectancy developed for this population.^6^ Accurate estimation of life expectancy at diagnosis is essential for WW. The European Association of Urology guidelines^2^ recommend using the European Union eurostat life expectancy table^21^ for this estimation, along with several other comorbidity measures, including the CCI. None of the previously reported studies on WW have considered any of these comorbidity measures. In this study, we combined the CCI with the DCI, which has been shown to outperform the CCI.^10^

The methodology used in this study consequently provided a 20-year follow-up using comprehensive information on PCa characteristics, treatments, patient age, and time-updated measures of comorbidity. In previous studies of men with PCa, this modeling approach accurately captured changes in treatment strategy and state transitions.^8,9^ In addition, we could verify good agreement between observed and simulated overall survival (eFigure 3 in Supplement 1). We have previously analyzed the robustness of estimates from state transition models using both sensitivity analyses and a comparative approach and found that these models provide stable estimates in terms of both calibration and prediction.^5,6,17,18^

Several clinical trials and observational studies have reported the outcomes for men with PCa treated with a WW strategy.^22^ In a frequently cited Swedish study of untreated early-stage PCa diagnosed in older men, most cancers had an indolent course during the first 10 to 15 years, and most men died of other causes.^4^ However, when survival exceeded 15 years, PCa mortality rose from 15 per 1000 person-years to 44 per 1000 person-years. Similarly, a cohort study from the US showed that men with low-risk PCa did not commonly die of PCa.^23^

Previous studies of WW included men with a life expectancy longer than 10 years. The Scandinavian Prostate Cancer Group Study Number 4 trial reported outcomes after 29 years of follow-up.^1^ Of men with clinically detected localized PCa in the WW group, 31% died of PCa, which was significantly higher than in curatively treated men. Fewer men older than 65 years at diagnosis, ie, with a shorter life expectancy, died of PCa (approximately 25%). A more contemporary study of men with PCa who were treated with observation is the Prostate Cancer Intervention Versus Observation Trial.^24^ At a median follow-up of 18 years, mean overall survival in the observation group was 12.6 years, vs 13.6 years in curatively treated men. This difference in favor of curative treatment was more pronounced in men younger than 65 years at diagnosis and having a better health status,^24^ again emphasizing the importance of baseline life expectancy. Of note, these historical series do not reflect the characteristics of contemporary patients^25^ due to both Gleason grade inflation and stage and grade migration.^26^

### Limitations

This study has some limitations. The transition from WW to CRPC is not recorded in the NPCR or in any other health care register in Sweden and, therefore, was approximated by use of a previously described algorithm.^8^ There may also be differences between countries and clinical settings in terms of diagnostic intensity, selection for WW, and treatment of men undergoing WW. Swedish guidelines have detailed recommendations regarding selection, treatment, and follow-up of these men.^27^ Adherence to these guidelines was recently shown to be good.^28,29^ The definition of WW in the NPCR is largely consistent with international guidelines.^30^

Another potential limitation is misclassification of the cause of death in older men. A recent Swedish patient record review showed that among men older than 85 years whose death was adjudicated to PCa in the Cause of Death Register, approximately 30% had moderate or no evidence of progression, irrespective of risk category.^31^ Therefore, the proportional risk of death from PCa could have been overestimated in our study and may explain why some of these deaths occurred before CRPC. The misclassification of cause of death may also indirectly bias the algorithm used to identify state transition to CRPC.

## Conclusions

In this cohort study, men with a nonmetastatic PCa who were treated with a WW strategy spent the largest part of their remaining lifetime without ADT. After 10 years, only a small proportion of men had transitioned to the CRPC state, and most deaths were attributable to causes other than PCa. These findings provide evidence that WW is an appropriate management strategy for minimizing adverse consequences of PCa in men with a baseline life expectancy of less than 10 years.

## Notes

Supplement 1. **eFigure 1.** CONSORT Diagram of Study Population **eTable.** Definition of Prostate Cancer Risk Categories **eMethods.** Detailed Description of State Transition Modeling **eFigure 2.** Possible Pathways Between Date of Castration (C+ADT) and Date of Censoring, Date of Death From Known and Unknown Cause **eFigure 3.** Observed and Simulated Survival Probability, Stratified Tertiles of Life Expectancy
eReferences

Supplement 2.
Data Sharing Statement
