# Supplementary material for: Natural History of Nonmetastatic Prostate Cancer Managed With Watchful Waiting
Source: JAMA Netw Open. 2024 Jun 4;7(6):e2414599. doi: 10.1001/jamanetworkopen.2024.14599 (PMC11151143; doi:10.1001/jamanetworkopen.2024.14599)
Supplement: Supplement 1. — eFigure 1. CONSORT Diagram of Study Population eTable. Definition of Prostate Cancer Risk Categories eMethods. Detailed Description of State Transition Modeling eFigure 2. Possible Pathways Between Date of Castration (C+ADT) and Date of Censoring, Date of Death From Known and Unknown Cause eFigure 3. Observed and Simulated Survival Probability, Stratified Tertiles of Life Expectancy eReferences [file jamanetwopen-e2414599-s001.pdf]

# Supplemental Online Content

Ventimiglia E, Gedeborg R, Styrke J, Robinson D, Stattin P, Garmo H. Natural History of nonmetastatic prostate cancer managed with watchful waiting. *JAMA Netw Open*. 2021;7(6):e2414599. doi:10.1001/jamanetworkopen.2024.14599

**eFigure 1.** CONSORT Diagram of Study Population

**eTable.** Definition of Prostate Cancer Risk Categories

**eMethods.** Detailed Description of State Transition Modeling

**eFigure 2.** Possible Pathways Between Date of Castration (C+ADT) and Date of Censoring, Date of Death From Known and Unknown Cause

**eFigure 3.** Observed and Simulated Survival Probability, Stratified Tertiles of Life Expectancy

**eReferences**

This supplemental material has been provided by the authors to give readers additional information about their work.

© 2024 Ventimiglia E et al. *JAMA Network Open.*

**eFigure 1. CONSORT Diagram of Study Population**

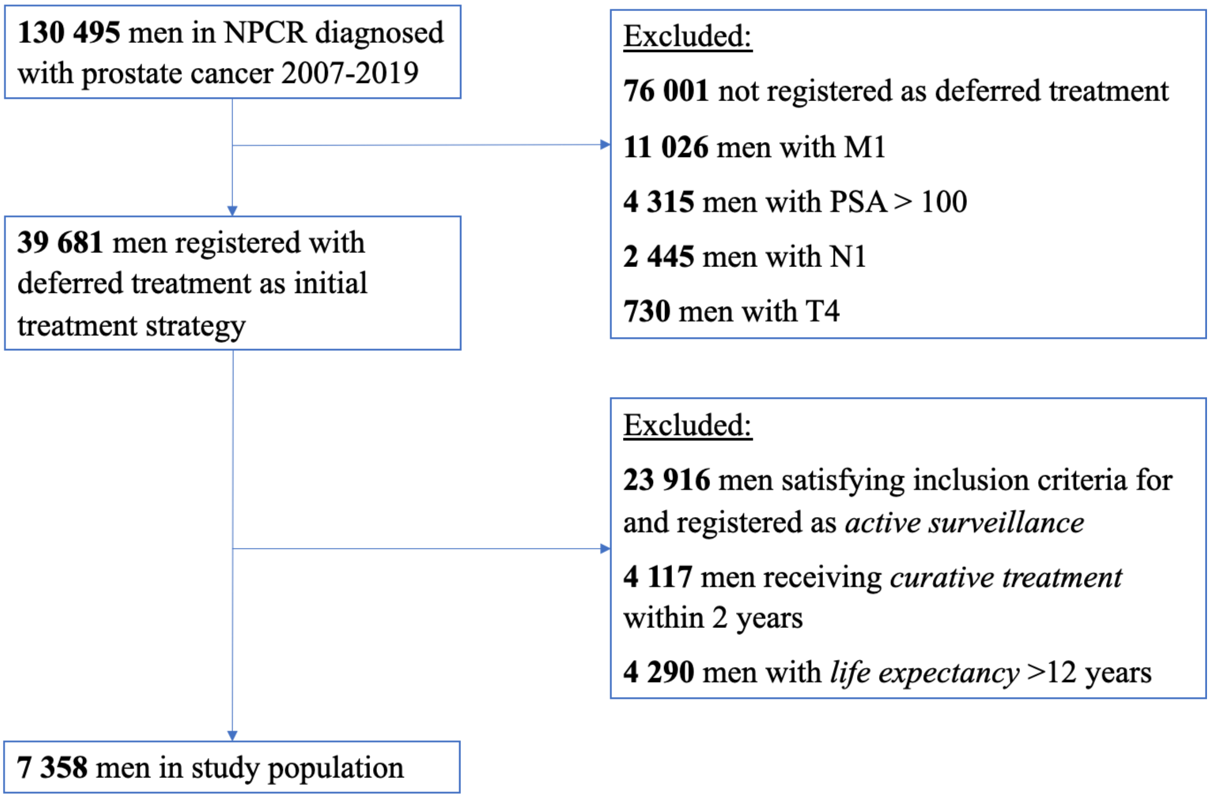


© 2024 Ventimiglia E et al. *JAMA Network Open.*

**eTable.** Definition of Prostate Cancer Risk Categories

| | **T-stage scoring**[a] | **PSA scoring**[b] | **Gleason score** |
|---|---|---|---|
| Risk categories | T1a = 1<br>T1b = 3<br>T1c = 2<br>T2 = 4<br>T3 = 6 | PSA≤20 = 1<br>20<PSA≤50 = 2<br>PSA>50 = 3 | 2-6 = 1<br>3+4 = 2<br>4+3 = 3<br>8 = 4<br>9-10 = 6 |
| Low risk | Total score ≤ 4 | | |
| Intermediate risk | Total score = 5-6 | | |
| High risk | Total score = 7 or higher | | |

© 2024 Ventimiglia E et al. *JAMA Network Open.*

**eMethods. Detailed Description of State Transition Modeling**

In accordance with the disease trajectory data management previously described, men registered as managed with watchful waiting but undergoing curative treatment within two years were not considered managed with watchful waiting. If treated curatively after two years this was considered as a censoring event.

To increase follow-up a microsimulation was performed for censored men. The simulation started on censoring date. Age and CCI at start of simulation was observed in data. At start of simulation each man was in either of the states watchful waiting, ADT, CSPC, or CRPC. For simulations starting in the watchful waiting-state the specific watchful waiting risk category was determined by TNM-stage, Gleason score and PSA as registered in NPCR. Simulations starting in any of the other states were based on the state specific risk category that was retrieved from the state specific ordinal regression models used in PCBaSe$^{Sim}$.

For men who were considered castrated (medically or by orchiectomy), i.e., in the CSPC-state, a conditional simulation was performed, conditioning on no death before censoring date. A list of all possible paths, consistent with the observed data (including all CCI-changes) was created, differentiating between different CSPC risk categories (see **eFigure 2**). The probability of each path was calculated using the transition probabilities specified in PCBaSe$^{Sim}$ including ordinal regression models used to determine risk categories. A randomly selected path was selected from the list of possible paths with a probability proportional to the path-specific probability. From this conditional simulation it was determined if the CRPC was reached or not prior to date of censoring. If so the CSPC risk category was extracted.

Similarly, for men who were not censored but followed to date of death a similar conditional simulation was performed, i.e., lists of all possible paths consistent with the observed data were created, probabilities of each path calculated, and randomly selected paths were chosen (see **eFigure 2**).

© 2024 Ventimiglia E et al. *JAMA Network Open.*

**eFigure 2.** Possible Pathways Between Date of Castration (C+ADT) and Date of Censoring, Date of Death From Known and Unknown Cause. CRPC, castration resistant prostate cancer, DP, death from prostate cancer. DO, death from other causes.

Date of

Date of

| | | | | | | |
|---|---|---|---|---|---|---|
| Path no 1 | C+ADT | C+ADT | C+ADT | C+ADT | C+ADT | C+ADT |
| Path no 2 | C+ADT | C+ADT | C+ADT | C+ADT | C+ADT | CRPC |
| Path no 3 | C+ADT | C+ADT | C+ADT | C+ADT | CRPC | CRPC |
| Path no 4 | C+ADT | C+ADT | C+ADT | CRPC | CRPC | CRPC |
| Path no 5 | C+ADT | C+ADT | CRPC | CRPC | CRPC | CRPC |
| Path no 6 | C+ADT | CRPC | CRPC | CRPC | CRPC | CRPC |

Date of

Date of

cause specific

| | | | | | | | |
|---|---|---|---|---|---|---|---|
| Path no 1 | C+ADT | C+ADT | C+ADT | C+ADT | C+ADT | C+ADT | DP |
| Path no 2 | C+ADT | C+ADT | C+ADT | C+ADT | C+ADT | CRPC | DP |
| Path no 3 | C+ADT | C+ADT | C+ADT | C+ADT | CRPC | CRPC | DP |
| Path no 4 | C+ADT | C+ADT | C+ADT | CRPC | CRPC | CRPC | DP |
| Path no 5 | C+ADT | C+ADT | CRPC | CRPC | CRPC | CRPC | DP |
| Path no 6 | C+ADT | CRPC | CRPC | CRPC | CRPC | CRPC | DP |

Date of

Date of

death with

| | | | | | | | |
|---|---|---|---|---|---|---|---|
| Path no 1 | C+ADT | C+ADT | C+ADT | C+ADT | C+ADT | C+ADT | DP |
| Path no 2 | C+ADT | C+ADT | C+ADT | C+ADT | C+ADT | CRPC | DP |
| Path no 3 | C+ADT | C+ADT | C+ADT | C+ADT | CRPC | CRPC | DP |
| Path no 4 | C+ADT | C+ADT | C+ADT | CRPC | CRPC | CRPC | DP |
| Path no 5 | C+ADT | C+ADT | CRPC | CRPC | CRPC | CRPC | DP |
| Path no 6 | C+ADT | CRPC | CRPC | CRPC | CRPC | CRPC | DP |
| Path no 7 | C+ADT | C+ADT | C+ADT | C+ADT | C+ADT | C+ADT | DO |
| Path no 8 | C+ADT | C+ADT | C+ADT | C+ADT | C+ADT | CRPC | DO |
| Path no 9 | C+ADT | C+ADT | C+ADT | C+ADT | CRPC | CRPC | DO |
| Path no 10 | C+ADT | C+ADT | C+ADT | CRPC | CRPC | CRPC | DO |
| Path no 11 | C+ADT | C+ADT | CRPC | CRPC | CRPC | CRPC | DO |
| Path no 12 | C+ADT | CRPC | CRPC | CRPC | CRPC | CRPC | DO |

© 2024 Ventimiglia E et al. *JAMA Network Open.*

**eFigure 3.** Observed and Simulated Survival Probability, Stratified Tertiles of Life Expectancy

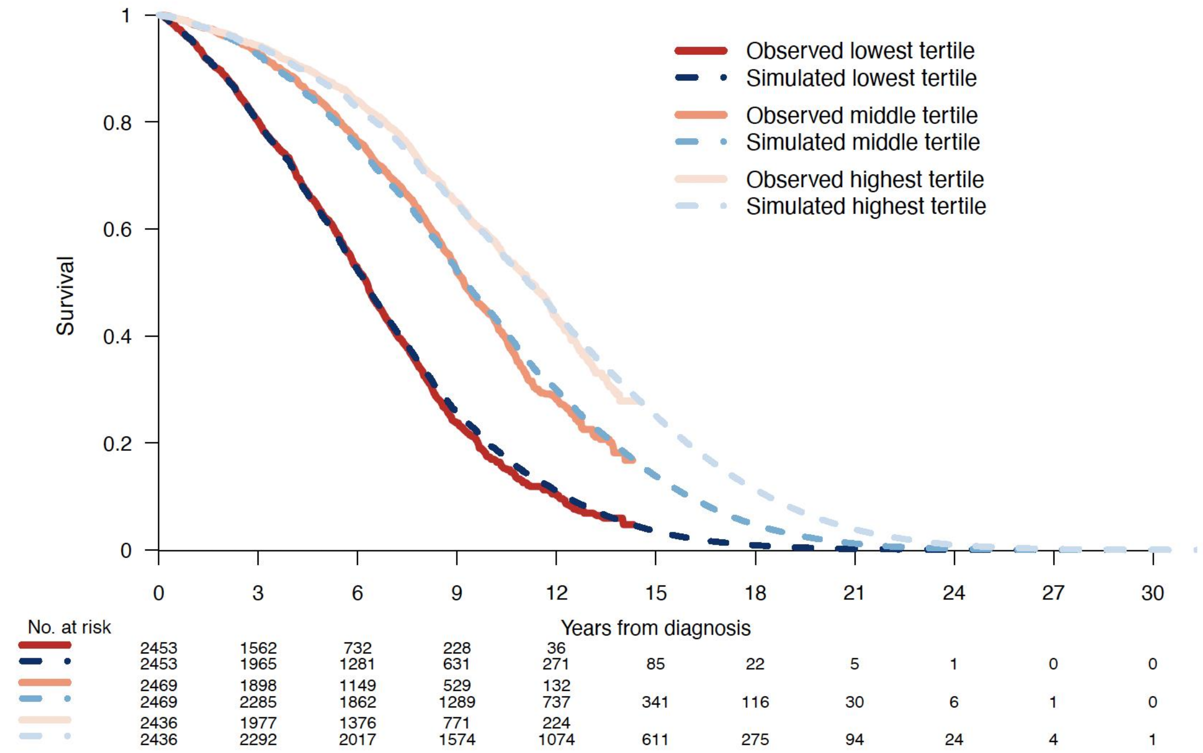


## eReferences


1. Charlson M, Szatrowski TP, Peterson J, Gold J. Validation of a combined comorbidity index. Journal of clinical epidemiology. 1994;47(11):1245-51.
2. Ludvigsson JF, Appelros P, Askling J, Byberg L, Carrero J-J, Ekström AM, et al. Adaptation of the Charlson comorbidity index for register-based research in Sweden. Clinical epidemiology. 2021;13:21.
3. Klabunde CN, Legler JM, Warren JL, Baldwin L-M, Schrag D. A refined comorbidity measurement algorithm for claims-based studies of breast, prostate, colorectal, and lung cancer patients. Annals of epidemiology. 2007;17(8):584-90.


© 2024 Ventimiglia E et al. *JAMA Network Open.*
