# Supplementary material for: Natural History of Nonmetastatic Prostate Cancer Managed With Watchful Waiting
Source: JAMA Netw Open. 2024 Jun 4;7(6):e2414599. doi: 10.1001/jamanetworkopen.2024.14599 (PMC11151143; doi:10.1001/jamanetworkopen.2024.14599)
Supplement: Supplement 2. — Data Sharing Statement [file jamanetwopen-e2414599-s002.pdf]

Data Sharing Statement

Ventimiglia. Natural History of Nonmetastatic Prostate Cancer Treated With Watchful Waiting. *JAMA Netw Open*. Published June 04, 2024. doi:10.1001/jamanetworkopen.2024.14599

Data
Data available: No

Additional Information
Explanation for why data not available: Data cannot be made available due to the Swedish law.
